# 

**Published:** 1879-05

**Authors:** 


### CONTENTS.

COMMUNICATIONS.

Historical Sketch of the Ohio College of Dental Surgery. *By Geo. Watt, M. D., D. D. S.* ........ 185
The New Departure and the Ideal "Filling." *By W. Mitchell, D. D. S., Delaware, O.* ........ 201

PROCEEDINGS OF SOCIETIES.

Mississippi Valley Dental Society ........ 204

MISCELLANEOUS.

Petermann's Dental Almanach for 1879 ........ 219

EDITORIAL.

Correction ........ 221
Josiah Bacon's Death ........ 221
Replanting Teeth ........ 223
Dental Legislation ........ 223
Commencement ........ 226
Alumni Association ........ 226
Illinois State Dental Society ........ 227
Transactions ........ 228

